# Synergistic Effect of Endogenous and Exogenous Aldehydes on Doxorubicin Toxicity in Yeast

**DOI:** 10.1155/2018/4938189

**Published:** 2018-05-30

**Authors:** Jana S. Miles, Samantha J. Sojourner, Aurellia M. Whitmore, Devon Freeny, Selina Darling-Reed, Hernan Flores-Rozas

**Affiliations:** College of Pharmacy and Pharmaceutical Sciences, Florida A&M University, 1415 S. Martin Luther King, Jr. Blvd. NPB Phase-2-L147, Tallahassee, FL 32307, USA

## Abstract

Anthracyclines are frequently used to treat many cancers including triple negative breast cancer, which is commonly observed in African-American women (AA), and tend to be more aggressive, carry worse prognoses, and are harder to manage because they lack molecular targets. Although effective, anthracyclines use can be limited by serious side effects and eventually the development of drug resistance. In* S. cerevisiae*, mutants of* HOM6* display hypersensitivity to doxorubicin.* HOM6* is required for synthesis of threonine and interruption of the pathway leads to accumulation of the threonine intermediate L-aspartate-semialdehyde. This intermediate may synergize with doxorubicin to kill the cell. In fact, deleting* HOM3* in the first step, preventing the pathway to reach the* HOM6* step, rescues the sensitivity of the* hom6* strain to doxorubicin. Using several* S. cerevisiae* strains (wild type,* hom6*,* hom3*,* hom3hom6*,* ydj1*,* siz1*, and* msh2*), we determined their sensitivity to aldehydes and to their combination with doxorubicin, cisplatin, and etoposide. Combination of formaldehyde and doxorubicin was most effective at reducing cell survival by 31-fold–39-fold (in wild type cells) relative to doxorubicin and formaldehyde alone. This effect was dose dependent on doxorubicin. Cotreatment with formaldehyde and doxorubicin also showed increased toxicity in anthracycline-resistant strains* siz1* and* msh2*. The* hom6* mutant also showed sensitivity to menadione with a 2.5-fold reduction in cell survival. The potential use of a combination of aldehydes and cytotoxic drugs could potentially lead to applications intended to enhance anthracycline-based therapy.

## 1. Introduction

Doxorubicin is one of the most effective anticancer agents [1]. Doxorubicin is an anthracycline antibiotic that is used to treat a variety of cancers including hematological cancers, carcinomas, and sarcomas [2–4]. This particular anthracycline antibiotic composes the major therapeutic alternative in breast cancer [5–7]. One of the three primary mechanisms of action for doxorubicin is its intercalation into DNA which directly affects transcription and replication [8]. The second mechanism of action is inhibition of topoisomerase II activity by stabilizing the DNA-topoisomerase II alpha complex, effectively preventing the religation portion of the ligation-religation reaction that topoisomerase II catalyzes [9]. Doxorubicin also generates free radicals as it cycles between its quinone and semi-quinone structures during metabolic reactions and thereby generates reactive oxygen species (ROS) [10].

Although extremely effective, anthracyclines are subject to drug resistance and deleterious side effects [11, 12]. Some tumor cells develop modifications that confer resistance to anthracyclines [13]. Tumor cells that have elevated levels of glutathione peroxidase are less affected by anthracycline generation of ROS [12, 14–16]. Another feature of cells that aid in doxorubicin resistance is decreased topoisomerase II activity [12, 14, 15]. Less topoisomerase II equates to less DNA double strand breaks. Lastly, some tumor cell populations manifest improved ability to repair DNA breaks; therefore, DNA replication proceeds uninhibitedly, and the tumor not only survives but continues to grow [12, 14, 15].

A major and currently insurmountable complication with anthracyclines use is the development of cardiomyopathy that can manifest following a single dose as early as 24 hours after exposure [12, 17] or many years later after successful treatment [12, 17]. Doxorubicin induced dilative cardiomyopathy and congestive heart failure is a serious and potentially fatal adverse effect. Dilative cardiomyopathy and the subsequent development of congestive heart failure (CHF) are refractory to common cardiovascular therapy [18]. The toxic injury to the heart after doxorubicin treatment is a result of doxorubicin mechanism of action, the generation of ROS [12, 18]. The increase in ROS due to doxorubicin treatment occurs with the redox cycling between the quinone and hydroquinone rings and carbonyl reduction of moieties within doxorubicin structure [19, 20]. The heavy production of ROS overwhelms the ability of antioxidizing enzymes to deal with them [19, 20].

Because doxorubicin effectively treats a wide variety of cancers [21, 22] and patient quality of life is improved when doxorubicin is included in the treatment regimen [23], significant efforts are being directed at discovering modalities to hypersensitize cells to doxorubicin [1, 24, 25]. We previously reported a genome-wide genetic screening in* S. cerevisiae* that identified 71 deletion strains displaying varying levels of sensitivity to doxorubicin. The screen revealed that inactivation of the HOM6 gene caused an accumulation of the L-aspartate-semialdehyde metabolite and increased the sensitivity of the* hom6* mutant to doxorubicin. To further investigate the contribution of the L-aspartate semialdehyde intermediate in the sensitization of cells to doxorubicin, we evaluated the survival of threonine biosynthesis mutant strains to doxorubicin. We extended this work by evaluating the sensitivity of different yeast deletions strains to various exogenously added aldehydes, either alone or in combination with doxorubicin and other cytotoxic stressors that mediate the action of these agents, such as oxidative stress, and DNA damage. Our results indicate that formaldehyde synergizes with doxorubicin to kill wild type* S. cerevisiae* cells and increases the sensitivity of doxorubicin resistant strains to doxorubicin.

## 2. Materials and Methods

### 2.1. General Genetic Methods and Strains

Homozygous haploid strains are all derived from the wild type parental strain (BY4741:* MATa his3Δ1 leu2Δ0 met15Δ0 ura3Δ0*). Specific deletion strains are* hom3, hom6, siz1, msh2, ydj1, rad52* and were obtained from Thermo Scientific (Pittsburgh, PA). The double knock-out strain,* hom3hom6,* was constructed by PCR mediated gene disruption of the* HOM3* gene in the* hom6* strain as previously described [8]. Yeast extract/peptone/dextrose (YPD, 1% yeast extract, 2% peptone, 2% dextrose, and 2% agar), yeast extract/peptone/glycerol (YPG, 1% yeast extract, 2% peptone, and 3% glycerol), or the corresponding drop-out media were as described in [6, 7]. Yeast strains were streaked initially onto YPG agar to eliminate petites, prior to growing in YPD for analysis. All incubations were carried out at 30°C [26, 27].

### 2.2. Chemicals

Yeast extract, peptone, and dextrose were purchased from Fisher Scientific (Fair Lawn, NJ); yeast nitrogen base was purchased from Thermo Scientific (Pittsburgh, PA, USA); doxorubicin-HCl (2 mg/mL) was obtained from Bedford Laboratories; formaldehyde (34.5%) was obtained from Amresco (Solon, OH); yeast media were purchased from Sigma-Aldrich (St. Louis, Mo). Menadione (Vitamin K3) was purchased from Enzo Life Sciences (Farmingdale, NY); etoposide was purchased from Chem-Impex Int'l. Inc. (Wood Dale, IL). Working solution concentrations were prepared as follows: doxorubicin (20 *μ*M) and formaldehyde (2 mM) were prepared in UltraPure sterile water, aliquoted, and stored at −20°C. Menadione (6.6 mM) and etoposide (0.5 mM) were prepared in appropriate solvent just before use.

### 2.3. Sensitivity of Strains to Aldehydes, Chemotherapeutic Agents, and Cytotoxic Stressors

The concentration of the drugs used for strain exposure was determined experimentally using the wild type parental strain, BY4741. Briefly, single colonies were grown overnight in liquid YPD media, at 30°C with shaking. Cells were then washed and resuspended in UltraPure sterile water. Strains were then separated into control and treatment groups and exposed to aldehydes alone and combined with cytotoxic chemotherapeutic agent for 3 hours. After exposure, cells were again washed and suspended in sterile water. 10-fold serial dilutions were spotted onto YPD agar plates and incubated at 30°C. Heat shock treatment was performed by plating serial dilutions of the strains and the plates were incubated at 37°C for 3 days. Cell growth was monitored daily and colonies were counted at day 3. Survival was calculated relative to the corresponding untreated control and sensitivity was determined relative to the survival of the wild type strain. All trials (3 minimum) involved testing independent colonies for each cytotoxic agent or stressor.

### 2.4. Statistical Analysis

Data analysis and graphing were performed using the GraphPad Prism 4 software package. Specific analysis for each experiment is indicated in each figure legend. In most cases, the mean of at least three experiments is plotted together with the standard deviation. Differences between mean values and multiple groups were analyzed by one-way analysis of variance (ANOVA). Statistical significance was set at *p* < 0.05.

## 3. Results

### 3.1. Defects in Threonine Biosynthesis Sensitize Cells to Doxorubicin

**Figure 1:**
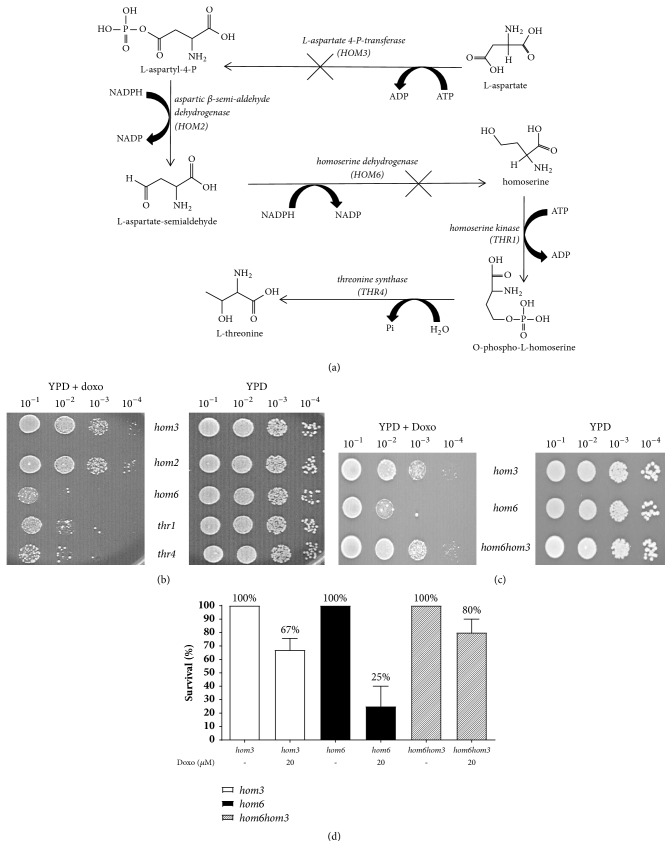
Defects in threonine biosynthesis sensitize cells to doxorubicin. Inactivated genes in the threonine biosynthetic pathway result in increased toxicity of doxorubicin (a, b). Inactivation of the* HOM3* gene rescues the* hom6* strain (c). Quantitation of* hom3*,* hom6,* and* hom6hom3* survival after doxorubicin exposure, 20 *μ*M (d).

To determine the cause of increased sensitivity to doxorubicin in genes involved in amino acid biosynthesis, we performed survival assays on mutant strains of genes required for threonine biosynthesis. The genes* HOM3*,* HOM2*,* HOM6*,* THR1*, and* THR4* encode for enzymes that catalyze the sequence of reactions in the threonine biosynthesis pathway (Figure 1(a)). As seen in Figure 1(b), the* S. cerevisiae *mutant strains* hom3*,* hom2*,* hom6*,* thr1*, and* thr4* were all individually inactivated and all show some sensitivity to doxorubicin when exposed to doxorubicin containing media (YPD + doxo) compared to the media lacking doxorubicin (YPD). The* hom6* strain was the most sensitive to doxorubicin with the subsequent strains* (thr1 and thr4)* in the pathway being slightly less sensitive than the* hom6* but more than the* hom3* and* hom2* (Figure 1(b)). To determine why the hypersensitivity occurred after the inactivation of the* HOM6* gene, which encodes for the enzyme homoserine dehydrogenase, we inactivated the upstream gene* HOM3* (Figure 1(c)). The* hom6* strain with the inactivated* HOM3* gene was denoted by* hom6hom3* and showed decreased sensitivity to doxorubicin when replica plated onto YPD + doxo media compared to the YPD media (Figure 1(c)). In Figure 1(d), the survival of the* hom3* strain decreased to 67% with 20 *μ*M doxorubicin from the 100% survival of the control (a 1.5-fold sensitivity increase). The treatment of the* hom6* strain with 20 *μ*M doxorubicin reduced the survival to 25% when compared to its control but the survival of the* hom6hom3* strain was reduced to only 80%. The sensitivity of the* hom3* and* hom6hom3* strains was increased by only 1.5- and 1.3-fold, respectively; however, the sensitivity of the* hom6* strain was increased to doxorubicin by 4-fold. Inactivation of the* HOM6* gene may have caused an accumulation of the toxic metabolite L-aspartate semialdehyde.

### 3.2. Treatment with Formaldehyde Increases the Sensitivity of Wild Type* S. cerevisiae* to Doxorubicin

**Figure 2:**
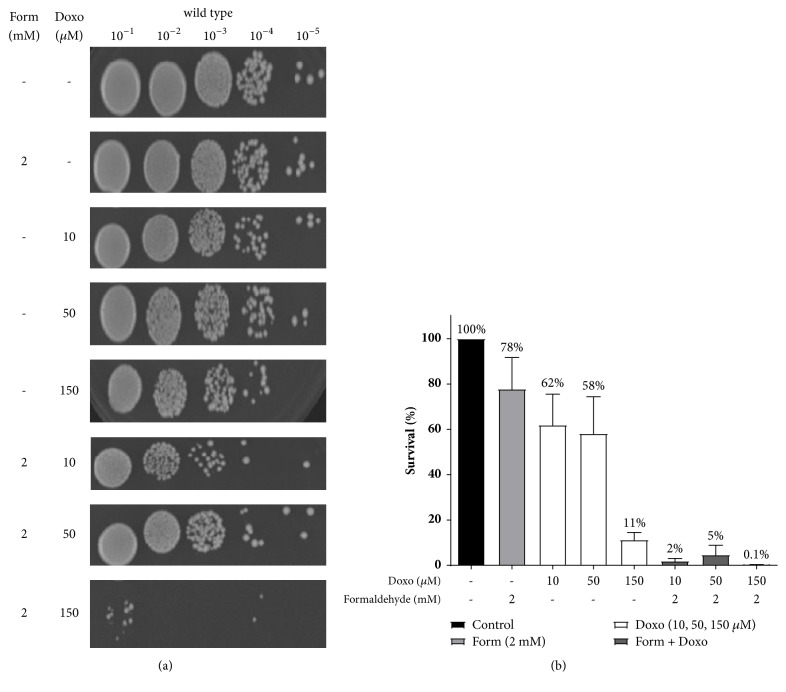
Formaldehyde synergizes with doxorubicin to kill wild type cells. (a) Growth of wild type cells tested by spotting onto YPD agar plates. (b) Quantitation of wild type cells treated with formaldehyde and doxorubicin, alone and in combination.

To explore the role of aldehydes on cells treated with doxorubicin, we used the wild type strain to determine if the aldehyde intermediate sensitized wild type cells to doxorubicin. As seen in Figure 2(a), when exposed to formaldehyde (2 mM) alone the strain shows some sensitivity, whereas when exposed to doxorubicin (10 *μ*M, 50 *μ*M, and 150 *μ*M) alone, the wild type strain showed dose-dependent reduction in growth (Figure 2(a)). The exposure of the wild type cells to the combination of formaldehyde (2 mM) and doxorubicin (10 *μ*M, 50 *μ*M, and 150 *μ*M) resulted in increased sensitivity relative to formaldehyde or doxorubicin alone (Figure 2(a)). Treatment with formaldehyde (2 mM) reduced the survival of the wild type strain to 78% (Figure 2(b)). However, in the presence of doxorubicin (10 *μ*M), formaldehyde reduced the viability of the strain by 50-fold relative to the untreated strain and around 31-fold and 39-fold relative to doxorubicin alone and formaldehyde alone, respectively (Table 1). Cotreatment of the wild type strain with formaldehyde (2 mM) and doxorubicin (150 *μ*M) reduces the viability of the strain by ~110-fold relative to formaldehyde alone. Our data shows that the cotreatment of formaldehyde (2 mM) and doxorubicin (10 *μ*M, 50 *μ*M, and 150 *μ*M) has a synergistic effect on the sensitivity of the wild type cells.

### 3.3. Effects of Cotreatment with Formaldehyde and Doxorubicin on Wild Type,* siz1*, and* msh2*

**Figure 3:**
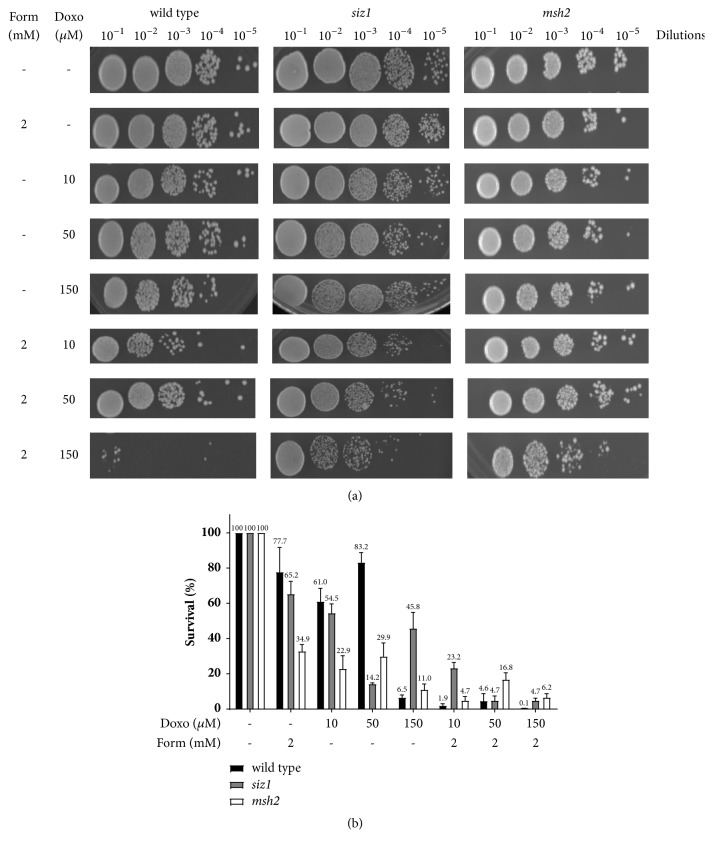
Cotreatment with formaldehyde enhances the toxicity of wild type and the doxorubicin resistant strains* siz1* and* msh2*. (a) Strains growth tested by spotting onto YPD agar plates. (b) Survival rates of strains following formaldehyde and doxorubicin treatment, alone and combined.

To further characterize the synergistic effect of formaldehyde and doxorubicin, a survival assay with cotreatment of formaldehyde (2 mM) and increasing concentrations of doxorubicin was performed in the strains* siz1* and* msh2*, which are deficient in septin sumoylation and mismatch repair, respectively. The* siz1 *and* msh2* strains were selected because previous studies showed that they are resistant to doxorubicin [8]. The* siz1* and* msh2* strains are indeed less sensitive to doxorubicin compared to the wild type (Figure 3) when subjected to the cotreatment of formaldehyde (2 mM) and increasing concentrations of doxorubicin. However, the increased toxicity of doxorubicin is apparent within each strain. The viability of the* siz1* and* msh2* strains was reduced most notably with formaldehyde (2 mM) and doxorubicin (10 *μ*M and 50 *μ*M) combined, than with either as a single treatment (Figure 3(a)). The survival of* siz1* after exposure to formaldehyde (2 mM) and doxorubicin (10 *μ*M) alone and combined was 65.2%, 53.9%, and 18.8%, respectively (Figure 3(b)). The survival of* msh2* after exposure to formaldehyde (2 mM) and doxorubicin (10 *μ*M) alone and combined was 34.9%, 22.8%, and 4.8%, respectively (Figure 3(b) and Table 2). Our data shows that the combination of formaldehyde and doxorubicin has a synergistic effect on wild type cells (≥31-fold increase) (Table 1); however, the effect is synergistic on* siz1* and* msh2* strains (Table 3). According to Table 3, the fold increase in sensitivity relative to formaldehyde alone is 3.5* (siz1)* and 5.2* (msh2)*. Although less pronounced than that of wild type, our data demonstrate that treatment of* siz1* and* msh2* strains with formaldehyde (2 mM) overcomes some of their resistance to doxorubicin.

### 3.4. The* hom6* Mutants Are Sensitive to ROS Generation

**Figure 4:**
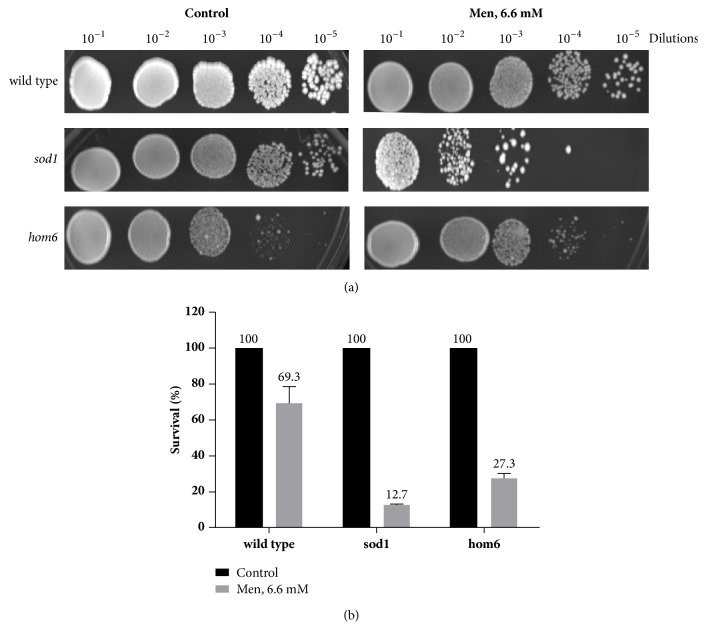
The* hom6* mutants are sensitive to the generation of ROS. (a) Growth of wild type,* sod1,* and* hom6* mutants after menadione (Men), 6.6 mM exposure. (b) Survival rates following Men, 6.6 mM exposure.

Menadione or vitamin K3 is a naphthoquinone derivative that is hepatically converted to menaquinone, an active form of vitamin K2. This agent was used in this study to determine the effect of increased ROS on the* hom6* mutant. The structure of menadione includes quinone ring [28] and, like doxorubicin, will induce oxidative stress by ROS generation [29]. Wild type,* sod1* (superoxide dismutase mutant), and* hom6* mutants were exposed to menadione, 6.6 mM (Figure 4(a)). The* sod1* strain served as the positive control because it cannot produce the antioxidant superoxide dismutase. As expected,* sod1* was the most sensitive to menadione induced ROS (12.7% survival) (Figure 4(b)). The survival of the* hom6* mutant was 27.3% (Figure 4(b)). The data shows that there was an increase in fold sensitivity relative to the wild type strain of 5.5 and 2.5 for the* sod1* and* hom6* mutants, respectively (Table 4). The results indicate that the* hom6* mutant is sensitive to the generation of ROS.

### 3.5. The* hom6* Mutant Was Not Sensitive to DNA Double Strand Breaks

**Figure 5:**
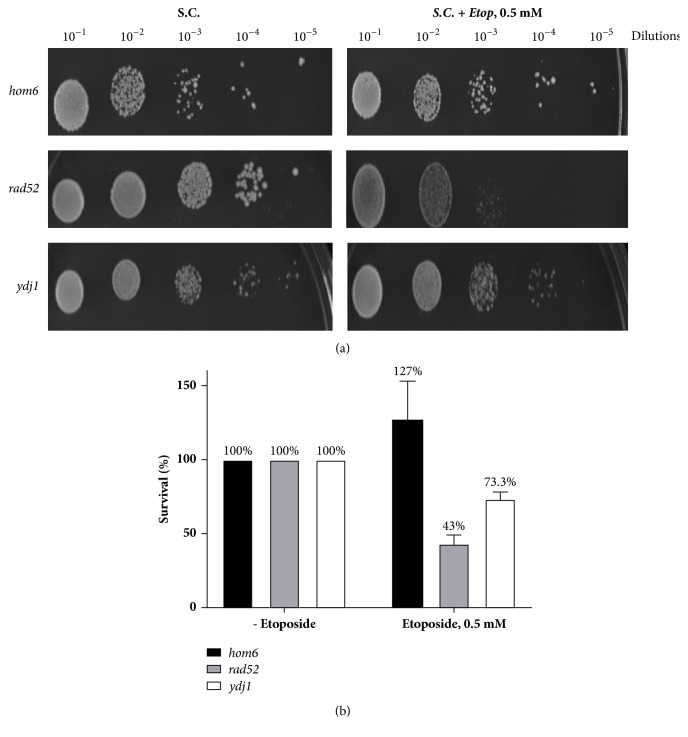
The* hom6* mutants are not sensitive to the generation of DNA double strand breaks. (a) Growth of* hom6*,* rad52,* and* ydj1* mutants after Etoposide (Etopo), 0.5 mM exposure. (b) Survival rates following Etopo, 0.5 mM exposure.

Etoposide is an antineoplastic compound that, like doxorubicin, binds to topoisomerase II and DNA and then induces DNA double strand breaks to kill the cell. The* hom6*,* rad52, *and* ydj1* mutants were spotted onto synthetic complete (S.C.) media with and without etoposide, 0.5 mM. The* rad52* (homologous recombination) and* ydj1* (protein repair) mutants grew less following etoposide exposure; however, the* hom6* mutant was not sensitive to DNA double strand breaks (Figure 5(a)). The data shows that* rad52* and* ydj1* survival were 43% and 73% after exposure to etoposide, respectively (Figure 5(b)). The fold sensitivity of the* rad52* and* ydj1* mutants relative to the wild type is 2.3 and 1.4, respectively (Table 5). The sensitivity of the* hom6* mutant is 0.8-fold relative to the wild type and is statistically insignificant (Table 5). This was expected because the* rad52* strain is unable to repair DNA damage by homologous recombination and the* ydj1* mutant is unable to repair protein damage; therefore, it is reasonable that they would be sensitive to a drug that induces DNA double strand breaks. However, the results indicate that DNA double strand breaks alone are unable to induce cell death in the* hom6* mutant.

## 4. Discussion

This study has demonstrated that the accumulation of L-aspartate semialdehyde enhances the toxicity of doxorubicin to the cell. Previous work in our laboratory identified a deletion of the* HOM6* gene which hypersensitizes cells to doxorubicin, suggesting that the threonine biosynthetic pathway can serve as a novel target for cell sensitization to cytotoxic chemotherapy [1]. Mutants of the* HOM6* gene confer significant increased sensitivity to doxorubicin. We extended this observation by investigating the role of aldehydes in the response of cells to anthracyclines.

We tested all the nonessential mutants of the threonine biosynthetic pathway (Figure 1) to determine which deletion strains exhibited higher sensitivity in response to doxorubicin. Three deletion mutants,* hom6*,* thr1,* and* thr4*, were found to be consistently hypersensitive to doxorubicin. It is worth emphasizing that the concentration of the drugs used is selected to identify hypersensitive strains. Increasing the dose will eventually kill all strains, including the wild type, and would not allow us to discriminate those hypersensitive strains from those unaffected.

The* HOM2* gene encodes for the aspartic beta semi-aldehyde dehydrogenase enzyme that produces the toxic metabolite L-aspartate-semialdehyde; this semialdehyde intermediate immediately precedes the strains in the threonine biosynthetic pathway which are hypersensitive to doxorubicin.

We tested the sensitivity of* S. cerevisiae* strains to the combination of formaldehyde (2 mM) and doxorubicin and our data showed that wild type cells are more sensitive to the combination of formaldehyde and doxorubicin than to either drug as a single agent (Figure 2). This sensitivity can be a result of the accumulation of the toxic aldehyde metabolite. Our data show that treatment with formaldehyde (2 mM) reduced the survival of the wild type strain to 78%. However, in the presence of doxorubicin, formaldehyde reduced the viability of the strain by 31-fold–39-fold (with 10 *μ*M doxorubicin) relative to doxorubicin and formaldehyde alone and by ~110-fold relative to doxorubicin at 150 *μ*M concentration. The cotreatment of formaldehyde (2 mM) and doxorubicin (10 *μ*M) has a synergistic effect in the wild type cells.

The* SIZ1* gene encodes the SUMO ligase enzyme which has been previously reported to have reduced accumulation of doxorubicin and therefore is slightly resistant to the effects of doxorubicin [1]. The* msh2* strain is deficient in mismatch repair and will therefore show higher survival relative to the wild type strain when exposed to doxorubicin [1]. In addition to the development of dose-dependent cardiomyopathy, another limiting aspect of doxorubicin therapy is drug resistance. The* siz1* and* msh2* strains were selected to determine if cells that have developed a resistance to doxorubicin therapy could be sensitized with the addition of an aldehyde. Cotreatment with formaldehyde (2 mM) and doxorubicin (10 *μ*M) of anthracycline-resistant strains* siz1* and* msh2* also shows increased toxicity (Figure 3). The cotreatment of formaldehyde and doxorubicin shows a toxic synergistic effect in* siz1* and* msh2* strains (Table 2). The combination at the lowest concentration of doxorubicin was just as effective as at higher concentrations at overcoming doxorubicin resistance in* siz1* and* msh2* cells (Figure 3). This would be advantageous in the development of protocols which would spare normal tissues from doxorubicin toxicity.

Since doxorubicin can act through the generation of ROS or the generation of DSBs, it is important to investigate which of these mechanisms is responsible for the synergistic effect between the L-aspartate semialdehyde and doxorubicin. Our strategy was to treat the* hom6* mutant with a drug that shares a single similar mechanism of action with doxorubicin at a time. The agents menadione and etoposide were employed for that purpose. Menadione or vitamin K3 is a naphthoquinone derivative that is hepatically converted to menaquinone, an active form of vitamin K2. Menadione has a quinone ring [28] and, like doxorubicin, will induce oxidative stress by ROS generation [29]. Etoposide is an anticancer agent and, similar to doxorubicin, binds to topoisomerase II and DNA. The stable union induces DNA double strand breaks while simultaneously preventing strand religation and subsequently leading to cell death. Our data showed that accumulated L-aspartate semialdehyde sensitized the* hom6* mutant to menadione induced ROS (Figures 4(a) and 4(b)) but not to etoposide (Figure 5) induced DNA double strand breaks. This suggests that the synergism between accumulated L-aspartate semialdehyde within the* hom6* mutant and doxorubicin is the result of combined mechanistic actions.

It has been reported that formaldehyde inhibits the cytochrome P450 enzymes CYP2C11, CYP2E1, and CYP3A2 while inducing the activity of the CYP1A2 enzyme [30]. Interestingly, the CYP3A family has been shown to metabolize doxorubicin and drugs that inhibit CYP3A results in elevated plasma concentration of doxorubicin [31]. It is also possible that formaldehyde coadministered with doxorubicin reduces the activity of CYP2C11 enzyme which results in elevated levels of doxorubicin [32–34].

Threonine is an essential amino acid; therefore humans must acquire this amino acid solely from dietary means. The effect of aldehyde combined with doxorubicin needs to be studied in nonessential amino acids that produce semi-aldehyde intermediates to further characterize the role of aldehydes in doxorubicin sensitization.

## 5. Conclusions

In conclusion, cotreatment of the wild type strain with formaldehyde and doxorubicin enhances the cytotoxic effect and reduces viability more than either as a single agent. Previous reports indicate that doxorubicin sensitive cancer cells have higher levels of endogenous formaldehyde compared to resistant cells that conversely lack elevated endogenous formaldehyde levels [35]. It was observed that wild type strain was sensitive to formaldehyde alone and to doxorubicin in a dose-dependent manner. However, the cotreatment of formaldehyde and doxorubicin on the wild type strain was significantly more cytotoxic than either formaldehyde or doxorubicin alone. The observed and calculated result indicated that formaldehyde synergizes with doxorubicin to kill wild type cells [36]. It has been reported that the central carbon in formaldehyde forms an adduct with DNA and doxorubicin and this adduct is proposed to be more toxic to cells than is doxorubicin [36]. The formaldehyde doxorubicin adduct could account for the synergistic effect seen with the cotreatment of formaldehyde and doxorubicin and this property can be exploited as a therapeutic enhancer. In fact, the pivaloyloxymethyl butyrate (AN-9) prodrug has been shown to be synergistic with doxorubicin and other anthracyclines [37]. The potential use of a combination of aldehydes and cytotoxic drugs may lead to applications intended to enhance anthracycline-based therapy by overcoming anthracycline drug resistance and reducing their toxic side effects.

## Figures and Tables

**Table 1:** Statistical analysis of wild type survival in response to formaldehyde and doxorubicin treatment.

Treatment		% Survival ± SEM	Expected value, %	Fold Sensitivity	*p* value
Form (mM)	Doxo (*μ*M)				
0	0	100	-	1	1
2	0	78 ± 13.9	-	1.2	0.2513
0	10	62 ± 13.6	-	1.6	0.038
0	50	58 ± 16.2	-	1.7	0.049
0	150	11 ± 3.2	-	9	<0.0001
**2**	**10**	**2 ± **1.1	**48**	50	0.0001
**2**	**50**	**5 ± **4.2	**45**	20	0.0019
**2**	**150**	0.1 ± 0.08	**8.5**	1000	<0.0001

**Table 2:** Formaldehyde and doxorubicin cotreatment enhances the toxicity of doxorubicin.

Strain	Formaldehyde (2 mM) Survival (%)	Doxorubicin (10 *μ*M) Survival (%)	Expected Additive Effect Survival (%)	Actual Effect Survival (%)
*msh2*	32.9	22.9	7.5	*6.3*
*siz1*	65.3	54.5	35.5	*23.2*
Wild type	77.7	61.9	48	*1.89*

**Table 3:** Statistical analyses of formaldehyde and doxorubicin relative to the actual percent survival. *p* value less than 0.05 is statistically significant.

	% Survival ± SEM	Relative to Form (2 mM) Alone		Relative to Doxo (10 *μ*M) Alone	
		Fold sensitivity	*p* value	Fold sensitivity	*p* value
*msh2*	6.3 ± 2.2	5.2	0.0090	3.6	0.0016
*siz1*	23.2 ± 2.8	3.5	0.0001	2.3	0.0004
Wild type	1.89 ± 1.1	41.0	0.0002	32.7	0.0004

**Table 4:** Statistical analysis of strain survival in response to Men, 6.6 mM. *p* value less than 0.05 is statistically significant. *p* value over 0.05 is not statistically significant.

Strain	% Survival ± SEM	Fold Sensitivity relative to wild type	*p* value
Wild type	69.3 ± 9.1	1	0.078
*sod1*	12.6 ± 2.7	5.5	<0.0001
*hom6*	27.3 ± 1.1	2.5	0.0014

**Table 5:** Statistical analysis of strain survival in response to Etopo, 0.5 mM. *p* value less than 0.05 is statistically significant.

Strain	% Survival ± SEM	Fold Sensitivity relative to untreated	*p* value
*hom6*	127 ± 15.1	0.8	0.21
*rad52*	43 ± 3.6	2.3	0.004
*ydj1*	73 ± 1.1	1.4	0.002

## Data Availability

Data will be available by contacting the corresponding author. All strains and reagents used in the studies are available upon request.
